# Supplementary figures and images for: Prediction of PM2.5 concentration based on the CEEMDAN-RLMD-BiLSTM-LEC model
Source: PeerJ. 2023 Aug 28;11:e15931. doi: 10.7717/peerj.15931 (PMC10470446; doi:10.7717/peerj.15931)

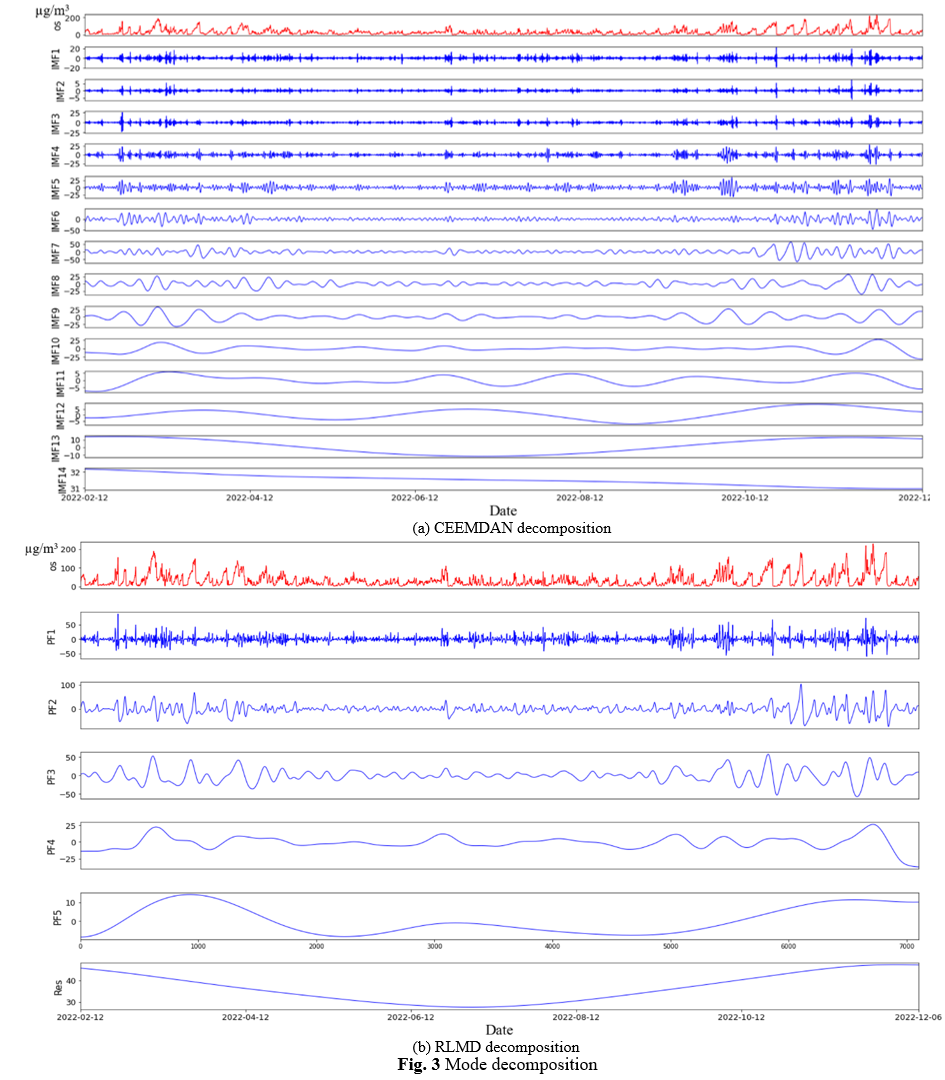

Supplement: Supplemental Information 1 [file peerj-11-15931-s001.png]

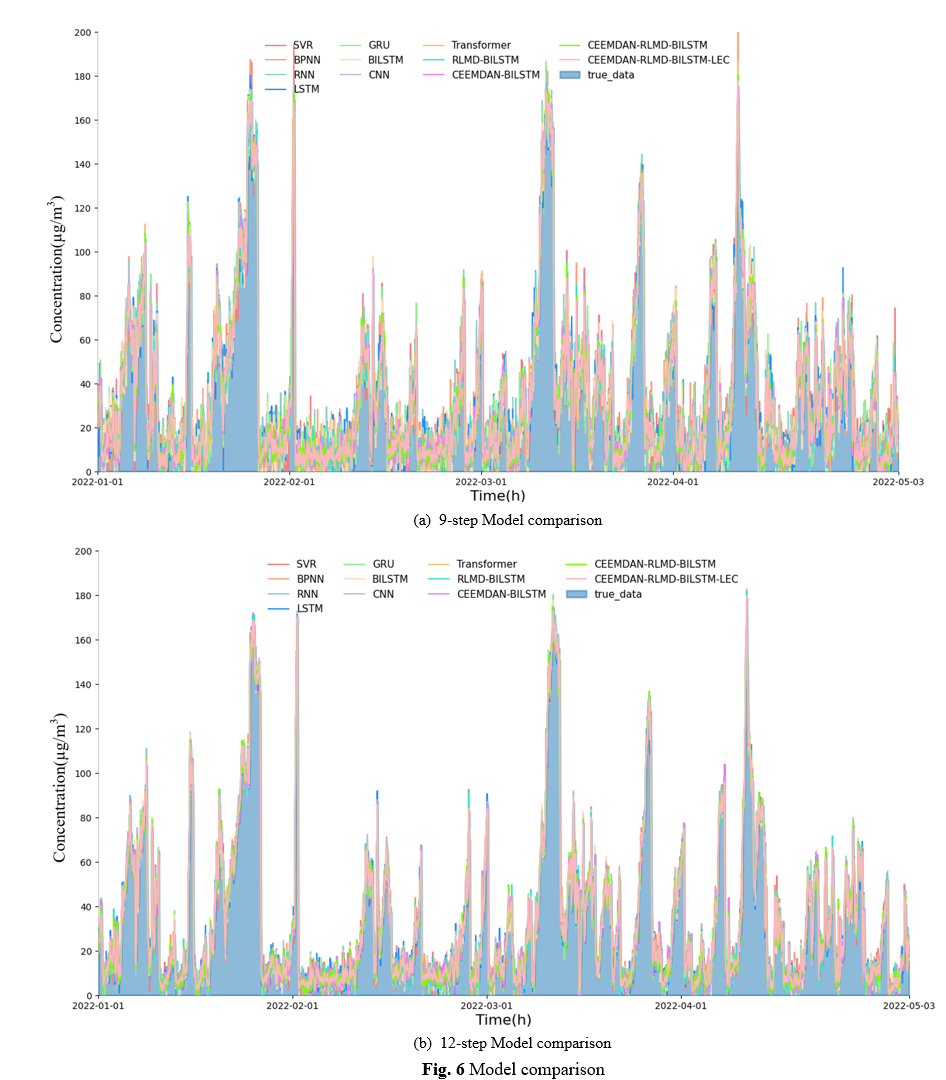

Supplement: Supplemental Information 2 [file peerj-11-15931-s002.png]

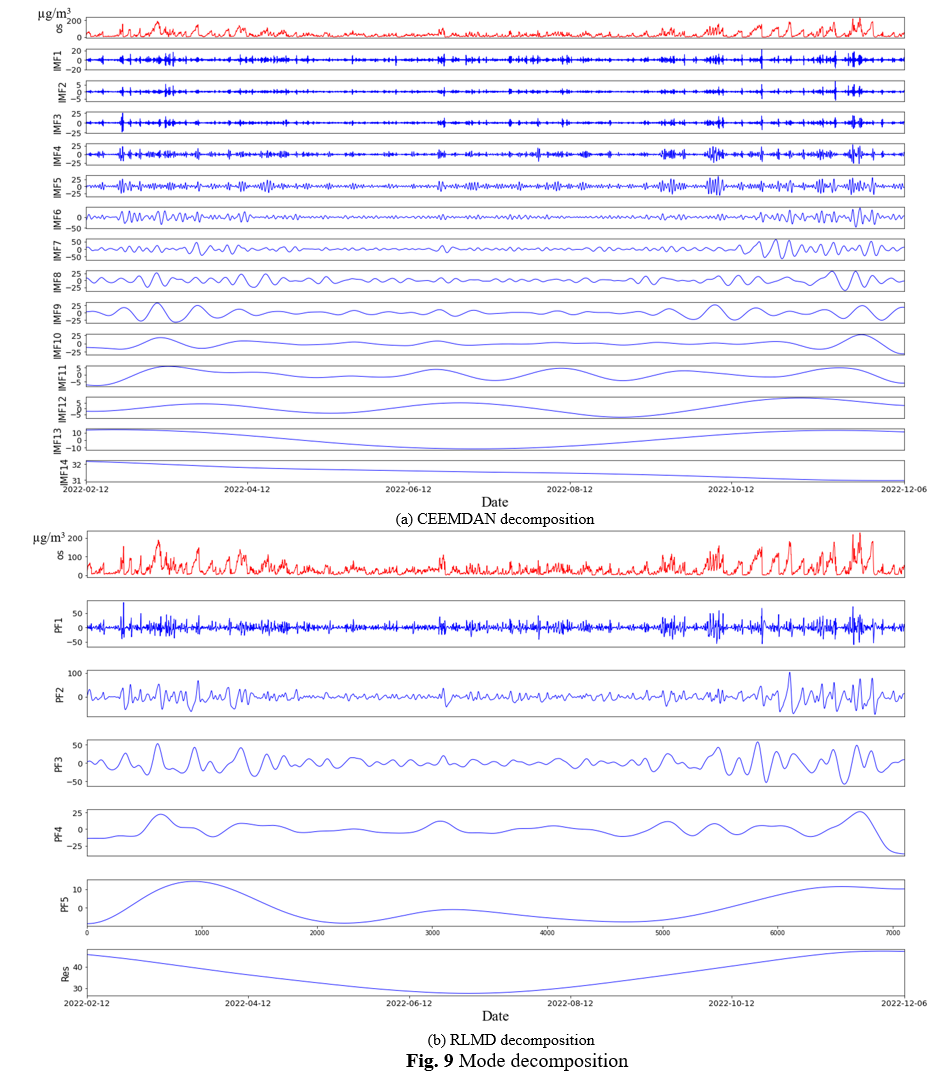

Supplement: Supplemental Information 3 [file peerj-11-15931-s003.png]

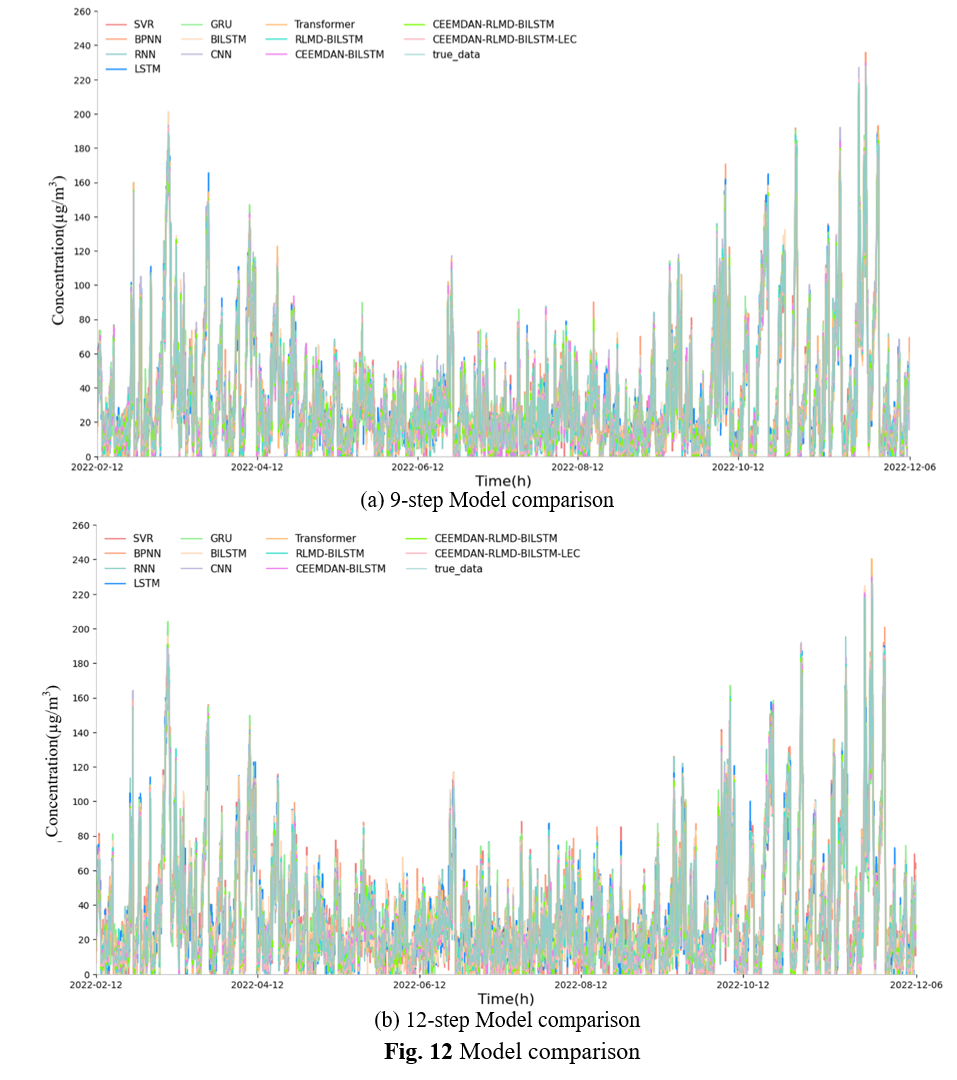

Supplement: Supplemental Information 4 [file peerj-11-15931-s004.png]
