# Supplementary material for: Prediction of PM2.5 concentration based on the CEEMDAN-RLMD-BiLSTM-LEC model
Source: PeerJ. 2023 Aug 28;11:e15931. doi: 10.7717/peerj.15931 (PMC10470446; doi:10.7717/peerj.15931)
Supplement: Supplemental Information 5 [file peerj-11-15931-s005.docx]

**Table 2** Multimodel prediction results

| **Steps length** | **Models** | **MAE** | **RMSE** | **SMAPE** |
| --- | --- | --- | --- | --- |
| 3-step | SVR | 24.1578 | 32.4475 | 0.7311 |
|  | BPNN | 20.1145 | 30.6221 | 0.6916 |
|  | RNN | 18.7789 | 25.9882 | 0.6288 |
|  | LSTM | 16.4255 | 24.8987 | 0.6126 |
|  | GRU | 23.1458 | 33.2110 | 0.7489 |
|  | BiLSTM | 15.0018 | 21.3204 | 0.5507 |
|  | Transformer | 4.0266 | 7.3258 | 0.1654 |
|  | CNN | 13.2214 | 21.6687 | 0.5897 |
|  | RLMD-BiLSTM | 6.5494 | 9.1135 | 0.2988 |
|  | CEEMDAN-BiLSTM | 5.0006 | 7.6690 | 0.2235 |
|  | CEEMDAN-RLMD-BiLSTM | 4.5005 | 7.6082 | 0.1807 |
|  | **CEEMDAN-RLMD-BiLSTM-LEC** | **3.6787** | **6.5912** | **0.1076** |
| 6-step | SVR | 20.1144 | 22.1475 | 0.6257 |
|  | BPNN | 17.5568 | 19.3354 | 0.5881 |
|  | RNN | 16.2144 | 18.1125 | 0.5591 |
|  | LSTM | 14.4071 | 18.1031 | 0.5398 |
|  | GRU | 19.4665 | 20.3365 | 0.6009 |
|  | BiLSTM | 10.8376 | 14.0857 | 0.4634 |
|  | Transformer | 5.0322 | 6.0984 | 0.1644 |
|  | CNN | 11.2254 | 14.9932 | 0.4877 |
|  | RLMD-BiLSTM | 7.1281 | 10.0532 | 0.3117 |
|  | CEEMDAN-BiLSTM | 5.3550 | 7.8676 | 0.2465 |
|  | CEEMDAN-RLMD-BiLSTM | 5.0541 | 7.2594 | 0.1907 |
|  | **CEEMDAN-RLMD-BiLSTM-LEC** | **4.0147** | **6.6374** | **0.1211** |
| 9-step | SVR | 16.3321 | 20.0114 | 0.6070 |
|  | BPNN | 13.1141 | 18.1223 | 0.5110 |
|  | RNN | 12.8611 | 16.8774 | 0.5001 |
|  | LSTM | 12.1001 | 16.3315 | 0.4911 |
|  | GRU | 15.1225 | 19.3365 | 0.5881 |
|  | BiLSTM | 9.4088 | 12.4936 | 0.4238 |
|  | Transformer | 5.5332 | 2.8951 | 0.0933 |
|  | CNN | 10.1124 | 13.2245 | 0.4324 |
|  | RLMD-BiLSTM | 6.7375 | 9.5696 | 0.2991 |
|  | CEEMDAN-BiLSTM | 5.1396 | 7.2770 | 0.2630 |
|  | CEEMDAN-RLMD-BiLSTM | 4.3013 | 6.8294 | 0.1637 |
|  | **CEEMDAN-RLMD-BiLSTM-LEC** | **3.4112** | **6.3411** | **0.0906** |
| 12-step | SVR | 24.1578 | 32.4475 | 0.7311 |
|  | BPNN | 20.1145 | 30.6221 | 0.6916 |
|  | RNN | 18.3365 | 28.3464 | 0.6741 |
|  | LSTM | 16.4255 | 24.8987 | 0.6126 |
|  | GRU | 23.1458 | 33.2110 | 0.7489 |
|  | BiLSTM | 9.8019 | 13.5730 | 0.4151 |
|  | Transformer | 5.3225 | 6.8977 | 0.1532 |
|  | CNN | 10.0212 | 13.8445 | 0.4533 |
|  | RLMD-BiLSTM | 6.5767 | 9.1802 | 0.2988 |
|  | CEEMDAN-BiLSTM | 5.1553 | 7.2870 | 0.2574 |
|  | CEEMDAN-RLMD-BiLSTM | 4.9244 | 7.2227 | 0.2207 |
|  | **CEEMDAN-RLMD-BiLSTM-LEC** | **3.9147** | **6.8155** | **0.1003** |

*Bold represents the optimal model.
