# Supplementary material for: Prediction of PM2.5 concentration based on the CEEMDAN-RLMD-BiLSTM-LEC model
Source: PeerJ. 2023 Aug 28;11:e15931. doi: 10.7717/peerj.15931 (PMC10470446; doi:10.7717/peerj.15931)
Supplement: Supplemental Information 6 [file peerj-11-15931-s006.docx]

**Table 3** Multimodel prediction results

| Steps length | Models | MAE | RMSE | SMAPE |
| --- | --- | --- | --- | --- |
| 9-step | SVR | 17.2682 | 19.0859 | 0.6307 |
|  | BPNN | 16.5643 | 17.7354 | 0.6069 |
|  | RNN | 14.2098 | 15.2976 | 0.5823 |
|  | LSTM | 13.6982 | 14.2843 | 0.5329 |
|  | GRU | 13.0943 | 15.3764 | 0.5427 |
|  | BiLSTM | 7.4938 | 9.2701 | 0.3921 |
|  | Transformer | 2.7556 | 3.9224 | 0.0727 |
|  | CNN | 5.9024 | 6.8146 | 0.1395 |
|  | RLMD-BiLSTM | 4.6689 | 7.2767 | 0.1072 |
|  | CEEMDAN-BiLSTM | 2.6778 | 3.7844 | 0.0577 |
|  | CEEMDAN-RLMD-BiLSTM | 2.5320 | 3.6558 | 0.0482 |
|  | **CEEMDAN-RLMD-BiLSTM-LEC** | **1.8299** | **3.0997** | **0.0389** |
| 12-step | SVR | 21.4237 | 24.2732 | 0.7212 |
|  | BPNN | 21.2774 | 23.6617 | 0.6847 |
|  | RNN | 20.0983 | 18.3068 | 0.6025 |
|  | LSTM | 19.2772 | 17.3736 | 0.5812 |
|  | GRU | 19.4326 | 17.5052 | 0.5709 |
|  | BiLSTM | 16.5889 | 13.0859 | 0.4237 |
|  | Transformer | 3.8221 | 6.4026 | 0.1451 |
|  | CNN | 11.9054 | 7.0172 | 0.1536 |
|  | RLMD-BiLSTM | 5.5920 | 7.7186 | 0.1061 |
|  | CEEMDAN-BiLSTM | 3.2012 | 4.3557 | 0.0682 |
|  | CEEMDAN-RLMD-BiLSTM | 2.6245 | 4.1023 | 0.0600 |
|  | **CEEMDAN-RLMD-BiLSTM-LEC** | **2.0934** | **3.4487** | **0.0459** |

*Bold represents the optimal model.
